# Examining comprehensive spatial and temporal amyloid deposition in brain and retina of hAbeta^SAA^ knockin mice

**DOI:** 10.1002/alz70856_107078

**Published:** 2026-01-08

**Authors:** Alaina M Reagan, Olivia J Marola, Michael Sasner, Gareth R Howell

**Affiliations:** ^1^ The Jackson Laboratory, Bar Harbor, ME, USA; ^2^ University of Maine, Orono, ME, USA; ^3^ Tufts University Graduate School of Biomedical Sciences, Boston, MA, USA

## Abstract

**Background:**

Developing methods for early detection of Alzheimer's disease and related dementias (ADRD) is critical for improved understanding of disease mechanisms and improved clinical outcomes. Annual optometrist or ophthalmologist visits commonly include non‐invasive, relatively inexpensive, image‐based assessment of retinal health. There has been conflicting data regarding the presence and measurement of amyloid deposition in the retina and its effectiveness as a biomarker. To address this, we are utilizing the hAbeta^SAA^ knock‐in mouse model that carries a humanized Aβ region of the mouse *App* gene, with Swedish, Artic, and Austrian mutations on a C57BL/6J (B6J) background. B6J mice homozygous for the Abeta^SAA^ allele develop human‐like amyloid plaque pathology and associated neuroinflammation without disturbing expression of the endogenous *App* gene. These data provide information about the utility of the retina as a biomarker for brain health.

**Method:**

Brains of B6J.hAbeta^SAA/SAA^ knockin (KI) mice develop amyloid plaques by 8 months of age, so we are assessing brain, retina and blood from KI and control B6J mice at 4, 8 and 12 months of age, allowing examination at timepoints that represent prodromal/early, mid and later stages of amyloid deposition. To provide alignment to common plasma‐based biomarkers, plasma Ab42/40 ratios are determined, as well as neurofilament light (NfL) and VEGF for neurodegeneration and vascular dysregulation respectively. Post‐mortem, presence of amyloid in brain and retinal tissue is detected using Congo Red, X‐34, ThioS, and Thiazen red. Additional glial and vascular stains assess support cell response to plaque development.

**Result:**

While detailed histological and immunostaining are underway for brain and retinal tissue at each timepoint, preliminary findings suggest dense core neuritic plaques are detected in the brain of B6J.hAbeta^SAA^ mice at earlier ages than in the retina. Alignment to plasma biomarkers are ongoing using mass spectrometry and ELISA.

**Conclusion:**

This comparative study will be the first to examine comprehensive spatial and temporal amyloid deposition in the aging retina and brain of the hAbeta^SAA^ model. Assessing amyloid using multiple staining methods will ensure we are able to detect all tissue‐specific peptides and plaques. Results will inform our use of the retina as a predictive tool for brain pathology.

